# An Observational Study to Evaluate the Usability and Intent to Adopt an Artificial Intelligence–Powered Medication Reconciliation Tool

**DOI:** 10.2196/ijmr.5462

**Published:** 2016-05-16

**Authors:** Ju Long, Michael Juntao Yuan, Robina Poonawala

**Affiliations:** ^1^ McCoy College of Business Administration Department of Computer Information Systems and Quantitative Methods Texas State University San Marcos, TX United States; ^2^ Ringful Health LLC Austin, TX United States; ^3^ Premier Physicians Austin, TX United States

**Keywords:** medication reconciliation, adverse drug event, medication errors, medication adherence, patient medication knowledge, decision making, shared

## Abstract

**Background:**

Medication reconciliation (the process of creating an accurate list of all medications a patient is taking) is a widely practiced procedure to reduce medication errors. It is mandated by the Joint Commission and reimbursed by Medicare. Yet, in practice, medication reconciliation is often not effective owing to knowledge gaps in the team. A promising approach to improve medication reconciliation is to incorporate artificial intelligence (AI) decision support tools into the process to engage patients and bridge the knowledge gap.

**Objective:**

The aim of this study was to improve the accuracy and efficiency of medication reconciliation by engaging the patient, the nurse, and the physician as a team via an iPad tool. With assistance from the AI agent, the patient will review his or her own medication list from the electronic medical record (EMR) and annotate changes, before reviewing together with the physician and making decisions on the shared iPad screen.

**Methods:**

In this study, we developed iPad-based software tools, with AI decision support, to engage patients to “self-service” medication reconciliation and then share the annotated reconciled list with the physician. To evaluate the software tool’s user interface and workflow, a small number of patients (10) in a primary care clinic were recruited, and they were observed through the whole process during a pilot study. The patients are surveyed for the tool’s usability afterward.

**Results:**

All patients were able to complete the medication reconciliation process correctly. Every patient found at least one error or other issues with their EMR medication lists. All of them reported that the tool was easy to use, and 8 of 10 patients reported that they will use the tool in the future. However, few patients interacted with the learning modules in the tool. The physician and nurses reported the tool to be easy-to-use, easy to integrate into existing workflow, and potentially time-saving.

**Conclusions:**

We have developed a promising tool for a new approach to medication reconciliation. It has the potential to create more accurate medication lists faster, while better informing the patients about their medications and reducing burden on clinicians.

##  Introduction

Medication error is one of the most common patient safety issues in the health care system [1-3]. Medication error is a major contributor to preventable adverse drug events (ADEs), which cause more than 3.5 million physician office visits, an estimated 1 million emergency department visits, and approximately 125,000 hospital admissions each year [4]. The national cost of ADEs is estimated to be $3.5 billion dollars every year [5]. Medication reconciliation is an intervention designed to reduce medication errors and ADEs. It is a process of creating the most accurate list possible of all medications that a patient is taking—including drug name, dosage, frequency, and route—and comparing that list against the existing medication list in the patient record [6].

Due to its early promise and large potential impact, medication reconciliation is mandated and reimbursed throughout the health care system. The Joint Commission specified medication reconciliation across the care continuum as a National Patient Safety Goal [7]. The Institute for Healthcare Improvement has medication reconciliation as part of its 5 Million Lives Campaign [8]. Medicare reimburses for medication reconciliation (Current Procedural Terminology code 111F) and has it as part of the requirement for Electronic Medical Record (EMR) Meaningful Use certification [9].

However, despite the high hopes, 2 large meta reviews revealed that medication reconciliation only had limited success in reducing ADEs in hospital settings [10,11]. Further investigations indicated that multidisciplinary team-based medication reconciliation approaches tend to work best. For example, a study showed a reduction of medication discrepancy from 89% to 49% by involving everyone, including patient, front desk staff, nurse, and physician, in the medication reconciliation process [12]. Specifically, 2 factors have emerged as important to medication reconciliation success. It includes the following:

1. Patient’s knowledge about his own medication use is of crucial importance for successful reconciliation [13]. In multiple studies, patients have demonstrated that they can identify discrepancies in their own medication lists with assistance from electronic tools [14,15].

1. The clinician team’s clinical knowledge gap is a major barrier for successful medication reconciliation [16]. For instance, nurses are inadequately trained on pharmacy subjects [17]. Pharmacist-led or nurse-pharmacist medication reconciliation demonstrated greater improvement in clinical outcomes [10,18,19]. It is also shown that providing specialized medication reconciliation training to medical residents could reduce medication discrepancy [20].

A promising approach to address both factors is to engage patients and supplement clinical knowledge using artificial intelligence (AI)-based electronic systems. The AI system can guide the patient to review his own medication lists and then to flag potential issues for the physician to review. For instance, the AI can understand hundreds of nonstandard abbreviations in handwritten prescriptions and can discern medications with multiple names that could confuse even expert clinicians [21]. It is shown that computerized systems have the capability to process medication terminologies [22].

The technology solution could also facilitate patient education by automatically showing the medication indications, side effects, prices, and other relevant information to patients. Such information is crucial for patients to make informed decisions on their medications. It could also flag potential discrepancies and prompt the patient to ask the clinicians in the care team to explain.

Electronic decision support tools have already shown promise in improving medication reconciliation [23-25]. A portable AI-powered decision support tool can not only help the patient identify and manage medications but also enable better team work because everyone can review the information on the screen together. Incorporating patients in the medical decision-making process has resulted in increased patient’s commitment and understanding of treatment plans, improved adherence, and increased patient satisfaction [26,27]. A recent survey indicated that patients themselves are very interested in using tablet devices in clinics to exchange information with the clinicians [28]. In primary care setting, having the patient do the work also has added benefits of saving clinician time and minimizing workflow interruptions.

In this paper, we will discuss how we built an AI-powered iPad tool to improve medication reconciliation and then evaluate the solution in real-world primary care settings.

##  Methods

###  Intervention

The software we developed for medication reconciliation is a Web-based app optimized for touch screen tablets, such as the Apple iPad. It takes a team approach to organize its workflow: The patient provides information on his up-to-date medication use and flags medications that he wants to discuss. The clinicians review the list together with the patient to determine what the most accurate list is. During the shared-screen review process, the clinicians can answer patient's questions about each medication and provide an opportunity to adjust certain medications to address patient concerns such as cost and side effects.

The app allows the front desk administrator to load a list of currently scheduled patients on the screen. The front desk administrator can tap on a patient name and then hand the tablet device to the patient at the time of check-in together with other medical or financial forms the patient needs to fill out.

#### The Use of AI Assistance

The intervention tool uses AI in 3 ways. First, it contains a machine learning (ML) module to recognize and parse prescription instructions written in natural language. The module is trained on over 2000 real-world prescription records from EMRs and achieved an error rate of less than 2%. Because all parsed medication records are again reviewed by both the patient and physician, the AI is truly an assistant and will not endanger patient safety. The details of the ML and natural language processing algorithms are beyond the scope of this paper.

Second, the tool aggregates multiple medication databases from National Institutes of Health, Food and Drug Administration (FDA), and commercial vendors to figure out equivalent or conflicting prescriptions in its medication reconciliation algorithm. The ability to organize data into knowledge representations and apply the knowledge to deduce meaning from real-world data is a hallmark of AI algorithms.

Finally, the tool can converse with the patient to go through the medication review process. The conversation is semistructured and rule-based.

#### Patient Workflow

The patient receives the tablet and confirms that his or her name is indeed on the screen. Following the screen instructions, the patient now starts the medication reconciliation process for the clinic visit.

**Figure 1:**
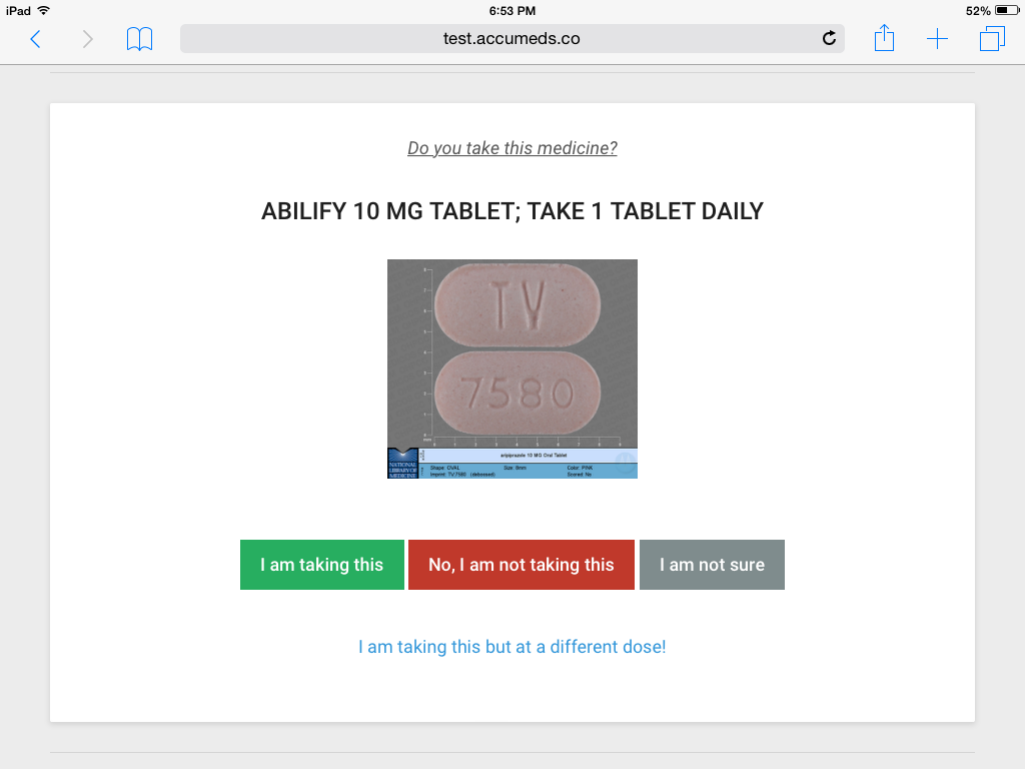
Review of a medication from the patient's list.

The patient’s current medication list is loaded by the app from the clinic’s connected EMR. The AI parses each natural language prescription record to break it into medication name, formulation, dose, and frequency information. The app will then present the medications to the patient one screen at a time (Figure 1). The screen shows the medication name, dose, instructions, and an image of the pill or package, if available. The patient has the options to do the following:

- Confirm that the patient is taking this medication as instructed.
- Remove the medication from the list because the patient is not taking it, or no longer taking it, or has not filled it.
- Mark the medication as uncertain if the patient is unsure whether he or she is taking it.
- Edit the strength and dose of the medication if those are incorrect.

**Figure 2:**
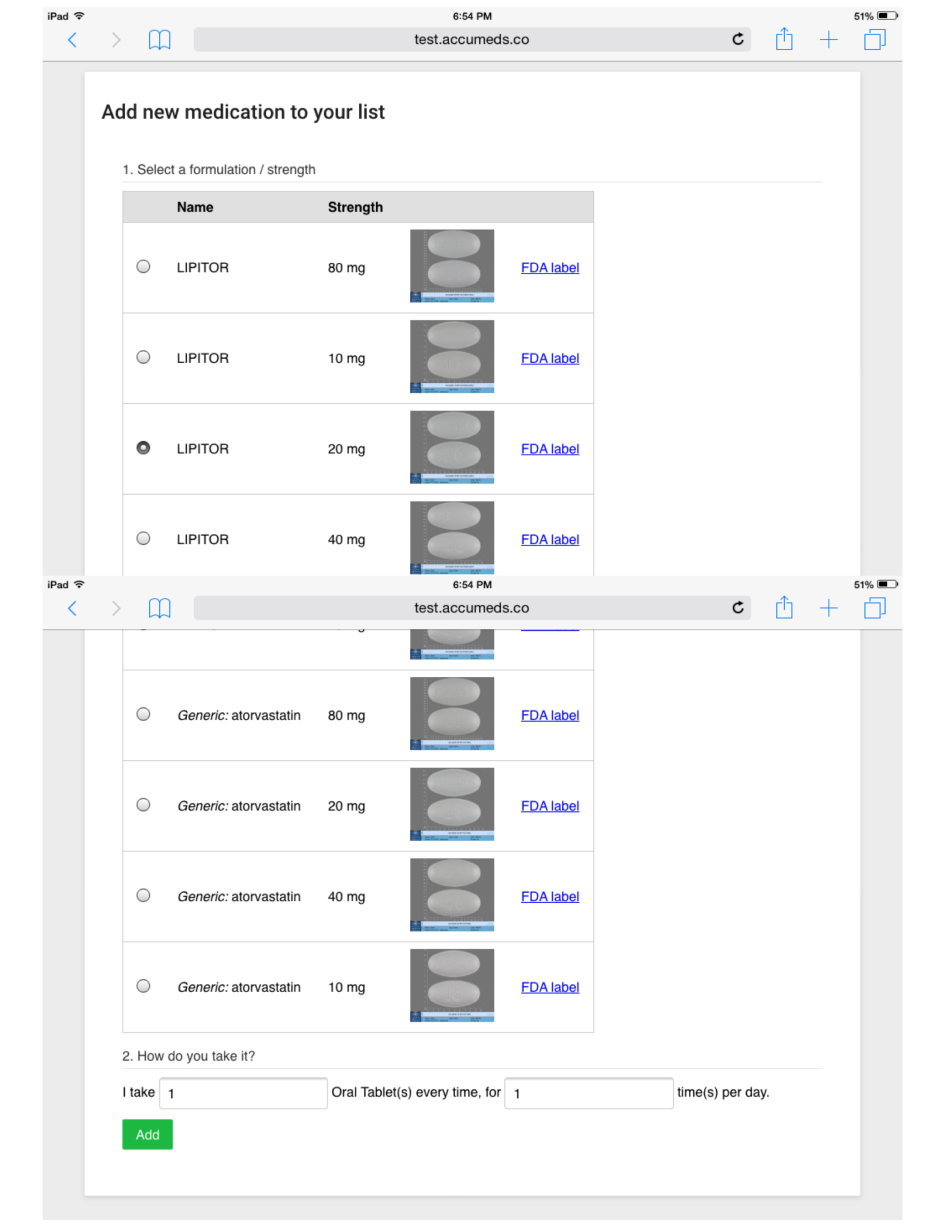
The process to add a medication to the list.

After the patient reviews all his or her current medications in the EMR, the app asks the patient if there are more to add. If the user chooses to add more medications, the app will show an auto-complete text field for the patient to add medications by just spelling out a few letters in the name. Once the patient chooses a medication name, the app lists all brand names and generic variations of this medication, with pill pictures if available. The user can select a medication from the list and then specify the number of pills taken every time and number of times the pills are taken per day. This process is depicted in Figure 2. The patient repeats this process until he or she has no more medications to add.

**Figure 3:**
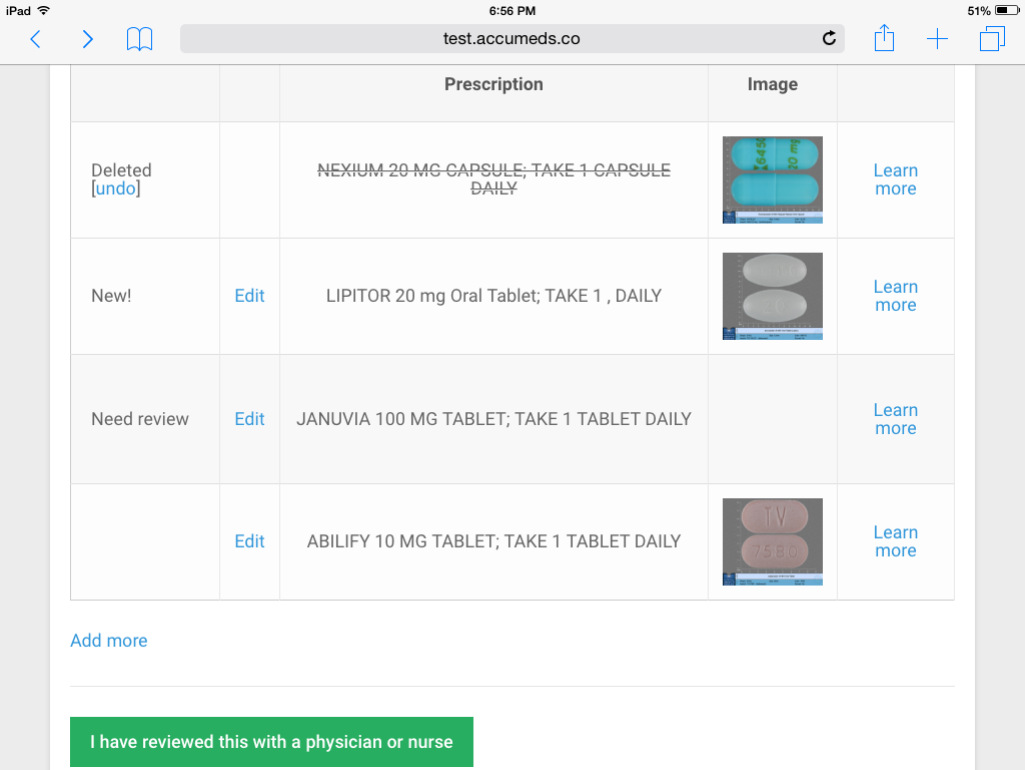
The reconciled medication list. This is the shared screen that the patient and clinician can work together to make and approve medication changes.

Once the patient is done, the app shows a “reconciled” list on the screen (Figure 3). When building this list, the AI goes through each medication, determines its generic active ingredients, and then compares them with other medications on the list. All duplicates will be automatically flagged by the AI. The AI-built reconciled list highlights medications that have been deleted, altered, or added. It also flags medications that the patient has marked as “unsure.” The patient can review the list for accuracy, and he or she can still add, delete, or make changes to any medication on the list. Notice that the screen shown in Figure 3 follows the patient into the clinical interview or encounter. The physician will work on this screen with the patient later.

After the patient is done reviewing his or her medication list, he or she might be still waiting to see the physician. The reconciled list provides a content page of educational material for each medication for the patient to learn more about his or her own medications, if the patient is interested. The educational page contains the following details:

- Link to FDA-structured label of the medication.
- Link to Medline Plus consumer content for selected medications.
- Link to GoodRx for selected medication so that the patient can look up cheapest prices for the medication in nearby local market.

Next, the patient brings the reconciled list on the tablet into the examination room. When the physician comes in, he or she will review the list with the patient together on the shared tablet screen (Figure 3). They will discuss why certain medications are stopped (eg, side effect, cost, and so forth) and make shared decisions on whether to change or discontinue certain medications. Once they are finished, the physician can close this screen and send the updated list back to the EMR.

After medication reconciliation, the clinical encounter happens as usual. The physician will examine the patient and potentially adjust medications based on new patient complaints. After the clinical encounter is over, the patient is likely to have an updated prescription. The patient will then follow on-screen instructions on the tablet to load the updated list from the EMR. The screen now shows the difference between the patient’s previous reconciled list and the new prescription list. It shows which medications are removed or added. The patient should now review this list and confirm whether it is indeed correct to his or her understanding. If the patient spots any issue at this time, he or she should request to clarify with the physician or a nurse before leaving the office.

The patient also has the option to access his or her medication lists from home. The patient will receive a secure message containing his or her login credentials in the clinic’s patient portal.

### Observational Study

To evaluate the usability of the solution, an observational study was conducted in a South Austin Family Practice clinic. We followed and observed 10 patients with complex medications over a span of 2 days. We examined whether they were able to complete the core medication reconciliation tasks on the tablet device and structured the observation using a software usability heuristic checklist.

The patients are selected by the office administrator the day before the study based on the current appointment schedule. The patient selection criteria are as follows:

- Each patient must have at least 5 active prescribed medications.
- The patient is previously scheduled to see Dr. Poonawala in the clinic on the 2 study dates.
- Medicare patients are prioritized for recruitment.

The selected patient is recruited at the time to check-in at the front desk. If the patient indicates that he or she is willing to participate, reads or writes English, and knows how to use a tablet device, the study coordinator will go over the informed consent with her. After the patient reviews and agrees to the consent, a tablet device will be provided to the patient with his or her personal and medical history information already loaded to start the study.

The study coordinator stays with the patient and passively observes how the patient is using the device. The study coordinator observes the patient’s interaction with the app and then notes any problems or difficulties the patient encounters. The patient issues are categorized according to 10 heuristics [29] commonly used to evaluate computer user interfaces.

### Patient Survey

In addition to the observational study conducted by a heuristic evaluator, the patient was directly asked at the end of the session how he or she perceives the usability of the product. We used the standard and widely used Computer System Usability Questionnaire [30] for this purpose. The patients answer those questions from SurveyMonkey Web site on the same tablet device they just used.

To evaluate the patients’ readiness to adopt and use the solution in future clinic visits, we surveyed them using a customized unified theory of acceptance and use of technology (UTAUT) instrument [31]. The UTAUT is designed to evaluate factors that could facilitate or impede future adoption of the technology. Our small sample of users does not have the statistical power to evaluate the entire model, but the survey answers gave us important suggestions on how to improve the product and deployment process to foster future adoption.

### Clinicians Interview

At the end of the clinical days, the researchers conducted one-to-one interviews with the physician and nurse who participated in the study. The one-to-one interview is unstructured [32], and it is designed to prompt free-form suggestions from the clinicians on how the tool impacted their clinical workflow. Specifically, we asked questions about whether the tool saved time for the clinicians and whether the clinicians feel the tool helped improve patient care or reduce potential medication errors.

The clinician responses were recorded in interview notes and are summarized in the Results section.

##  Results

The medication reconciliation tool is successfully deployed in the clinic and used by both patients and clinicians in the 2 days. In this section, we report results from the observation study, patient survey, and clinician interviews.

### Observational Study

#### Medication Reconciliation

We collected valid data from 10 patients in the study. The patients have the following characteristics:

- Between 50 and 87 years old.
- Seven are women, and 3 are men.
- Have between 5 and 16 active medications each before the appointment.

The 10 patients have a combined 92 active medications before the appointment. After review, they changed 26 medication records, representing 28% of the total records. All the patients changed at least 1 medication record. This indicates widespread problem in medication discrepancy and highlights the patient’s essential role in providing accurate medication information.

After the initial medication review and consultation with the physician, only 1 patient identified problem with the new prescription list at discharge. This indicates that the reconciliation has successfully brought the patient and clinicians on the same page with regard to medications.

#### Learning About Medications

Although the software tool provides extensive materials for the patient to learn more about their medications, including indications, side effects, and prices, no patient has taken advantage of these features. It appears that the clinic environment is not well suited for reading long articles and research reports. Several patients remarked that short video clips will be much more appealing while they wait.

In addition, patients have also indicated that they are more likely to read about their medications after they go home. This indicates the need to follow-up with the patient with email or other content after they go home to complete the patient education cycle.

#### Heuristic Evaluation of the User Interface

Key usability problems identified in the heuristic evaluation are categorized by the heuristics (Table 1).

**Table 1:** Heuristics of the software usability.

Heuristics	Examples
Aesthetic and minimalist design	The blank space for missing pill images is a waste of space and could be confusing to some users. The text boxes for dose or frequency in “add medication” are inconsistent with the rest of the UI.
Consistency and standards	The “confirm” action button colors and locations are inconsistent—it could be blue or green and could be located to the left or right.
Documentation and help	There is minimum in-app documentation or help available.
Error prevention	If the user taps on a wrong button while reviewing medications, there is no easy way to correct it. The user has to wait until the review screen, and the steps to correction are difficult.
Flexibility and efficiency of use	Adding medication to the list is inefficient. The user needs to figure out terms he or she is not familiar with, such as dose and frequency.
Help user recognize, diagnose, and recover from errors	When the user accidentally hits the home button, the iPad exits to the home screen without an obvious way to go back into the app. The user could force exit the app and lose the session. Some of the “invalid input” alert boxes are poorly worded.
Match between system and the real world	The delay in Web page loads mismatches the user experience in reviewing real-world paper-based forms.
Recognition rather than recall	Abbreviations are sometimes used in the text description.
User control and freedom	It is difficult to go back a few screens to correct a prior error.
Visibility of system status	There are often delays when the next Web page loads. During that time, the system appears unresponsive. Need to give strong visual clues for the “wait” status.

### Patient Survey

#### Usability

Almost all patients saw themselves as "not good at technology" and were initially uneasy about using the iPad. However, all the patients were able to use the core features of the tool with minimal help.

On the scale from 1 to 7 (7 being the most easy-to-use), the patients rated the tool a 6.5—very easy to use. The user satisfaction score for the tool is 6.0 of 7. Patients strongly agreed that the tool is a good idea (score 6.5 of 7).

#### Intent for Future Adoption

The patients showed strong interests in using the tool for future medical visits. When the patients were asked whether they will use the tool again in this clinic, 9 of 10 patients answered *Yes*. Of 10 patients, 8 answered that they will use it in a different clinic. However, only 5 of 10 patients would use the app to manage medications at home. We dictate that this reflects the user’s perception of limited utility for medication management at home without professional help nearby.

The tool is thought to be easy-to-use and have high perceived usefulness. However, at least 1 patient questioned the value of the tool to patients. She remarked that the tool seems to save time for clinicians but does not save time for the patient because the patient now needs to do more work. We dictate that this objection can be mitigated by emphasizing the tool’s benefit to patients in terms of reducing potential medication errors and harms.

Seven of 10 patients indicated that having someone to help in the clinic is a critical factor for them to adopt the tool. It is also true that the patient can more easily act on the results from the tool inside a clinic because the prescribing physician is on hand to review the results and potentially make changes to the prescription.

### Clinician Interview Results

The general consensus is that the tool saved the nurse time by reducing their questions about patient medications. However, making changes to medication on the tool is harder than doing it on paper. But, that is probably a reflection of the nurses being unfamiliar with the tool with only 10 patients using it.

During the pilot, the tool does not save time for the physician although increased familiarity with the tool could result in time-saving in the future. The physician reported more confidence in the accuracy of the patients’ medication list reported by the tool.

### Conclusions

The tablet-based medication reconciliation tool in medical clinic is well accepted by patients and clinicians. The AI component facilitated the patients themselves to recognize their own medications and report discrepancies for the clinicians to review. It has potentials to improve medication accuracy and reduce medication errors in the clinic.

## Abbreviations

AI: artificial intelligence
ADEs: adverse drug events
EMR: electronic medical record
ML: machine learning
UTAUT: unified theory of acceptance and use of technology
